# Metallurgical Waste Landfills as Potential Anthropogenic Deposits

**DOI:** 10.3390/ma19163447

**Published:** 2026-08-14

**Authors:** Katarzyna Nowińska, Aleksandra Czajkowska, Jarosław Sikorski

**Affiliations:** 1 Faculty of Mining, Safety Engineering and Industrial Automation, Silesian University of Technology, ul. Akademicka 2, 44-100 Gliwice, Poland; aleksandra.czajkowska@polsl.pl; 2 Division of Geochronology and Environmental Isotopes, Institute of Physics—Centre for Science and Education, Silesian University of Technology, ul. Konarskiego 22B, 44-100 Gliwice, Poland; jaroslaw.sikorski@polsl.pl

**Keywords:** metallurgical wastes, slags, lead, zinc, copper, iron, steel, landfill, deposits, resources

## Abstract

Metallurgical slags produced in the zinc–lead, copper, iron and steel metallurgical industries are characterized by a variety of technical parameters and varying chemical and mineral compositions, which depend on the type of raw material and the technological process selected and its workflow. These slags are characterized by a high content of valuable elements, including Cu, Pb, Zn, and Fe, which form complex multiphase conglomerates. In the chemical composition of Zn-Pb smelting slags, the contents of the dominant elements, i.e., Fe, Zn, and Pb, range from a few to several dozen %. These elements occur mainly in the form of polyphase oxides and silicates. The chemical composition of Cu smelting slag contains up to a few % Cu and up to several dozen % Fe, with these elements mainly forming silicates and sulphides. Iron and steel metallurgy slag contains up to several dozen % Fe, which occurs mainly in the form of silicates, oxides, and spinels. Due to their phase composition, metallurgical slags stored in landfills can have a negative impact on the environment. The phase composition of slags is one of the main factors determining their behavior in a weathering environment, i.e., their ability to release metals when exposed to atmospheric factors such as precipitation or temperature. On the other hand, such high concentrations of elements in metallurgical slags mean that they become anthropogenic deposits, and the phase composition of the slags determines how the deposited slags are processed. The aim of this article is to present the mineralogical and chemical characteristics of Zn, Pb, Cu and steel metallurgical slags deposited in landfills, along with an assessment of these materials as a source of secondary raw materials.

## 1. Introduction

The extraction and processing of mineral resources are of vital importance to the modern economy and enable the continuous development of our civilization. However, these processes result in a wide range of by-products, namely, various types of waste, mainly industrial. One of these is metallurgical slag generated during the smelting of metal ores or secondary raw materials, such as used batteries, scrap metal, and printed circuit boards [1].

Among the many classifications of slag, the most common is based on the type of ore processed in the metallurgical process. According to this classification, a distinction is made between, amongst others, slags produced during the processing of iron ore and steel production and non-ferrous slags [1,2].

Based on data on global iron and steel production, it has been estimated that between 530 and 690 million tons of slag were produced in 2021 [1,3], and recent commodity reports indicate that these significant production volumes remain a persistent global trend [4].

Slag from non-ferrous metal processing is produced in significantly smaller quantities than iron and steel slag. Based on data on total production of these metals in recent years, it is estimated that the annual production of copper and lead slags amounts to approximately 20.5 and 5.5 million tons, respectively [5].

European legislation treats non-ferrous and ferrous slags in a similar manner. According to the waste classification in [6], all slags fall under category 10, i.e., waste from thermal processes. Most slags (iron, steel and copper) are considered non-hazardous waste, but some (zinc–lead slags) are classified as hazardous waste. This is mainly due to the high concentration of metallic elements in the latter and the possibility of these being released into the environment as a result of weathering [1]. In addition, if they are not properly handled on the dump site, this might cause severe pollution with fine fractions of PM1 and PM2.5, which can easily induce silicosis, lung asthma and cancer.

A specific category is constituted by slags produced during the refining of raw lead, which are classified as hazardous waste and disposed of in landfills. An example of this is the refining slag that is produced during the pyrometallurgical (firing) process, which was used as the basis for the raw material analysis presented in this article.

Slags produced during the lead refining process are characterized by high levels of metals and metalloids; therefore, the landfill site where they are selectively deposited can be regarded as an anthropogenic deposit. The landfill is granted the status of an anthropogenic deposit when the following conditions are met: the collected material (e.g., mining waste, ash, slag) has the properties of a mineral resource, the quantity and quality of the material justify its potential extraction, the construction of the landfill and the types of substances stored there have been formally identified and documented.

From an economic perspective, it is important to assess the size of the deposit in order to classify it as an anthropogenic deposit, thereby enabling an evaluation of the viability of its exploitation. Although numerous reviews have discussed metallurgical waste management, the unique contribution of this paper lies in integrating mineralogy, element occurrence state, and environmental risk assessment to evaluate these landfills holistically as artificial anthropogenic deposits. For iron and steelmaking slags in particular, this integrated approach bridges the gap between viewing highly basic, free-lime-containing wastes merely as environmental liabilities and systematically redefining them as valuable raw materials for civil engineering and secondary metal recovery.

Previous review papers have generally focused on individual aspects of metallurgical waste, such as waste generation, chemical composition, recycling technologies or environmental impacts, usually considering individual slag types separately. In contrast, the present review adopts an integrated perspective by linking metallurgical processing, chemical and mineralogical composition, element occurrence state, environmental behavior and resource potential within a single conceptual framework applicable to different metallurgical waste streams. This approach enables the identification of common mechanisms governing both environmental stability and secondary resource recovery, thereby providing a comprehensive basis for assessing metallurgical waste landfills as anthropogenic deposits rather than merely as industrial waste disposal sites.

In addition, a new feature of this review article is its discussion of a specific type of slag from Zn-Pb smelting, namely, slag produced after lead refining.

Furthermore, this review compares zinc–lead, copper, and iron/steel slags using a unified evaluation framework, allowing for direct comparison of their mineralogical characteristics, environmental behavior and resource potential.

## 2. Characteristics of Metallurgical Waste

### 2.1. Zn-Pb Metallurgical Slag

Metallurgical slags, produced during the zinc and lead smelting processes, are characterized by a wide range of technical parameters, as well as chemical and mineral compositions, which depend on the type of feedstock, the technological process employed, and the course of that process. Given that metallurgical slags constitute one of the most diverse groups of waste materials, they should be treated on a case-by-case basis.

Refining slags have been classified as hazardous waste; therefore, when disposed of in a landfill, they may pose a potential threat to the environment, particularly to soil and water.

On the other hand, as refining slags contain numerous strategic and scarce metals, they can serve as a valuable source for their extraction.

#### 2.1.1. Waste-Generation Technologies (Metallurgical Process)

Slag from Zn-Pb smelting is produced during pyrometallurgical and hydrometallurgical processes.

Pyrometallurgical methods for Zn and Pb involve recovering metals from ores, concentrates or waste materials (recycling) through smelting at high temperatures.

The main advantage of these methods are their ability to recover zinc and lead simultaneously, which significantly improves the process’s economic efficiency [7,8,9,10].

The wastes generated during this process include slag (from the shaft furnace and refining), dust, sludge, and dross.

The product of the pyrometallurgical process is raw lead, which undergoes a fire-refining process, which is designed to purify raw lead by removing impurities and recovering associated metals, particularly precious metals. The refining process utilizes the properties of impurities, such as solubility, which decreases with a decrease in temperature (Cu and intermetallic phases), the ability to form insoluble intermetallic phases in lead (Ag and Bi), and a higher chemical affinity for oxygen (Sn, As, Sb, Zn, Ca, Mg), to separate them from lead. Due to the process conditions, the slag produced in this furnace contains significantly higher concentrations of metals compared to slag from a shaft furnace.

Due to the large quantities of slag generated in pyrometallurgical processes, they are the subject of this article.

Hydrometallurgical methods involve leaching roasted concentrate with a solution of H_2_SO_4_. The zinc sulphate solution obtained from the leaching process is purified to remove impurities, after which the zinc is recovered from the purified solution in electrolytic cells. The waste generated during this process consists mainly of filter sludge. These methods enable the production of high-quality metal, with a significantly lower environmental impact compared with smelting methods. However, these methods cannot be used for the production of lead, as the salts of this metal (PbSO_4_) are poorly soluble [7,8,9,10].

#### 2.1.2. Chemical and Phase Composition and Element Occurrence State

Slag from Zn-Pb smelting (shaft furnace slags) contains numerous elements, including Zn, Pb, Cu, Sb, As and Tl, which, although they mainly form sulphide and metallic phases, the dominant phase constituents of the slags are silicates, which are characterized by low leachability in an aqueous environment and, thus, do not pose a potential threat to the environment [2,11,12,13,14].

The chemical and phase composition of slags from Zn-Pb smelting depends on the type of feedstock, i.e., zinc–lead concentrates (of the deposit type), materials added to the process (e.g., fluxes) and process conditions.

Slag from the Zn-Pb metallurgical industry contains a number of components in its chemical composition, the predominant ones being Fe_2_O_3_ (0.88–59.6% wt.), SiO_2_ (2.04–57.1% wt.), CaO (0.18–32.23% wt.), Al_2_O_3_ (0.9–21.9% wt.), MgO (0.61–15.9% wt.), ZnO (0.03–47.3% wt.), PbO (0.0002 to 6.23% wt.) [2,15,16,17,18].

The phase composition of slags from Zn-Pb smelting is highly variable [2,16,18,19,20,21,22,23,24,25,26,27,28,29,30,31,32,33,34,35].

The main phase constituents of slag are as follows:

− Silicates and aluminosilicates (willemite (Zn_2_SiO_4_); olivines (Mg, Fe, Mn); SiO_4_, including fayalite (Fe_2_SiO_4_), kirschsteinite (CaFe^2+^SiO_4_) and forsterite (Mg_2_SiO_4_); pyroxenes (XY(Si,Al)_2_O_6_, where X = Ca, Na, Fe^2+^, Mg and Y = Cr, Al, Fe^3+^, Mg, Mn, Ti); and melilites (Ca,Na)_2_(Al,Mg) [(Si,Al)_2_O_7_]);
− Oxides and hydroxides (zincite (ZnO), wüstite (FeO), hematite (Fe_2_O_3_), and goethite (FeO(OH)));
− Sulphides (sphalerite (ZnS), galena (PbS), pyrite (FeS_2_), pyrrhotite (FeS), digenite ((Cu,Fe)_9_S_5_), cubanite (CuFe_2_S_3_), covellite (CuS), chalcocite (Cu_2_S), and sulphates and hydrated sulphates (anglesite (PbSO_4_), goslarite (ZnSO_4_·7H_2_O), gypsum (CaSO_4_·2H_2_O), rapidcreekite (Ca_2_(SO_4_)(CO_3_)·4H_2_O), ktenasite (ZnCu_4_(SO_4_)_2_(OH)_6_·6H_2_O), and posnjakite (Cu_4_[(OH)_6_|SO_4_]·H_2_O)), which usually form complex conglomerates or multiphase intergrowths).

In addition, slags contain carbonates (cerussite (PbCO_3_), smithsonite (ZnCO_3_), hydrozincite (Zn_5_[(OH)_3_/CO_3_]_2_), hydrocerussite (Pb_3_(CO_3_)_2_(OH))), metal alloys (Pb, Zn, Cu, Fe, As, and Sb) and pure metals (Zn, Pb, Cu, and Fe).

Another group of minerals (phases) commonly found in non-ferrous metal slags are spinels, mainly magnetite. The other phases belonging to this group identified in the slags are hercynite (FeAl_2_O_4_), franklinite (ZnFe_2_O_4_), gahnite (ZnAl_2_O_4_) and ulvöspinel (Fe_2_TiO_4_).

In the slags produced by Zn-Pb smelting, alongside crystalline components, there is an amorphous substance–glass—the quantity of which depends on the rate at which the slags are cooled. The glass content in slags cooled rapidly with water is significantly higher than in slags cooled slowly, e.g., in air [14].

#### 2.1.3. Environmental Risks

The primary criterion for classifying Zn-Pb slag as hazardous waste is its environmental impact, determined based on the results of leaching tests (dynamic and static) that simulate natural slag weathering processes or conditions at a landfill site. These tests make it possible to determine the mobility of the elements contained in the slag and to predict their long-term (even up to several hundred years) behavior in the environment.

Studies conducted by numerous authors have shown a significant degree of leaching of elements from metallurgical slags, with some of these elements subsequently being incorporated into secondary minerals formed under hypergenic conditions, which significantly limited their mobility [36,37].

The elements released from slag in the greatest quantities and characterized by significant solubility (>2.0 mg/L) are zinc, lead, and iron. Zinc leaching occurs over a long period of time; it has been found that after 12 years, the concentration of zinc in the eluate exceeds the permissible limit of 200 mg/kg for hazardous waste by a factor of 40 [38].

Under the pH conditions (4.5–6.0) of a hypergenic environment, the main forms of zinc are Zn^2+^, ZnCO_3_^2−^, and, less commonly, ZnSO_4_·7H_2_O. The mobility of zinc in near-neutral environments is limited, because it is readily adsorbed by oxides, hydroxides, and aluminosilicates [2].

The rate of lead leaching from slag as a function of time (after 12 years), despite its high solubility in water (>2 mg/L), is significantly lower than the rate of zinc leaching. This is due to the fact that the release of Pb is partially limited by the formation of new phases, such as carbonates and lead sulphates (including cerussite PbCO_3_ and anglesite PbSO_4_), which are insoluble in water. The increased mobility of lead is largely influenced by the salinity of the surface water layer, which promotes the formation of soluble complexes such as PbCl_2_, PbCl_4_^−^, and PbCl^+^.

The main source of iron in a hypergenic environment may be the release of Fe^2+^ and SO_4_^2−^ ions resulting from the oxidation of pyrotite in that environment. As geochemical modeling indicates, the dominant iron phases in near-surface conditions are melanterite (FeSO4), FeSO4·7H_2_O, goethite (FeO(OH)), ferrihydrite (Fe_5_HO_8_·4H_2_O), jarosite (KFe^3+^_3_(OH)_6_(SO_4_)_2_), and soluble iron sulphate [11].

Research conducted by Bril et al. (2008) showed that the sulphate phases of Cu, Pb, and Zn, such as brochantite (Cu_4_(SO_4_)(OH)_6_), anglesite (PbSO_4_), and bianchite ((Zn,Fe)SO_4_·6H_2_O), are secondary phases associated with the weathering of Zn-Pb slags in Poland [23]. The presence of the minerals listed above indicates the release of trace elements from anthropogenic phases (e.g., cynkite, melilite, spinels, enamel) during the weathering process in quantities sufficient to allow for the formation of secondary phases.

#### 2.1.4. Resource Utilization

Domestic waste disposal sites for the Zn-Pb smelting industry are located in the southern part of Poland; these are mainly historic sites, e.g., Bytom, Piekary, Sławków, Świętochłowice. It is estimated that historical Zn-Pb metallurgical waste landfills contain between several hundred thousand and over one million Mg of zinc, with an average Zn content in these landfills from 3% to 7% by weight of waste, and tens of thousands Mg of lead, and the average Zn content in these landfills is from 3% to 7% by weight of waste. Copper reserves in historical deposits, where it is present as an impurity, also amount to thousands of metric tons, with an average grade of less than 0.5% by weight of waste.

The only active zinc–lead smelting waste landfill in Poland is the hazardous waste landfill of the zinc smelter in Miasteczko Śląskie. The zinc and lead reserves in the refining slag within the landfill were estimated at 2856 and 5206 tons, respectively [8].

Since slags from lead refining are classified as hazardous waste, the best way to dispose of them is by recycling. The choice of technology for recovering metals from metallurgical waste, including slag, depends mainly on its mineralogical and chemical composition. Given the variety of forms in which metals occur in slag, it is extremely difficult to identify the optimal processing method.

Metals (Zn, Pb, and Cu) from slags produced during the pyrometallurgical process of Zn-Pb production are recovered using pyrometallurgical and hydrometallurgical methods.

The pyrometallurgical process (fire metallurgy) involves the processing of slags at high temperatures in various types of furnaces, including shaft, muffle, rotary, and electric furnaces [9,10].

One of the commonly used pyrometallurgical methods for processing smelting waste is fuming, which is mainly used to recover lead from slags generated during lead production that contain 2–25% Zn and up to 5% Pb. This process involves blowing a mixture of carbon powder and air through nozzles located in the walls of a cyclone furnace or converter at a temperature of 1200 °C into the molten slag.

Currently, the second-most widespread pyrometallurgical method is the remelting of lead smelting slag in Isasmelt- and Kaldo-type furnaces. These furnaces are used to process both primary and secondary raw materials [39]. The design of the Isasmelt furnace and the use of a steel lance make it possible to carry out melting, oxidation, and reduction processes. The remelting process is conducted at a temperature of 1150–1250 °C [39,40,41].

Hydrometallurgical methods for recovering metals from waste include leaching, solution purification, metal separation, and the recovery of pure metals at temperatures below 100 °C.

The leaching process, which forms the basis of hydrometallurgical processes, can be carried out using acid solutions (such as sulfuric or hydrochloric acid), ammonia solutions, and alkali hydroxide solutions. The choice of leaching reagent depends on the chemical form in which the metal is present in the waste [39,40,41].

Another group of methods used to recover metals from waste is bioleaching, a process in which microorganisms convert solid insoluble metals and their compounds into water-soluble forms [42].

Bioleaching can be used not only to recover valuable metals but also to remove toxic elements by breaking down the amorphous structure of slags.

The effectiveness of bioleaching depends on many factors, including pH, temperature, leaching time, and slag structure [42].

### 2.2. Waste from Cu Smelting

Copper smelting, particularly when based on pyrometallurgical processes, generates significant quantities of solid waste. Pyrometallurgical processes, including the smelting of ore concentrates, converter smelting and electrolytic refining, result in the production of copper slag and related materials (dusts and sludges), which are often deposited in landfills. Due to their volume, complex chemical composition and content of valuable metals, these wastes can be regarded as potential anthropogenic deposits [43]. It is recognized in the literature that waste from copper smelting is of great significance both from the perspectives of resource management and environmental protection [44,45,46,47,48]. Due to the presence of heavy metals and toxic elements (including Pb, Cd, As, Cu, and Zn) in copper slag that pose threats to the soil and groundwater environment, as well as living organisms, it is becoming increasingly necessary to reduce their landfilling by implementing sustainable processing strategies as part of a circular economy [11,25,45,48,49,50,51,52].

#### 2.2.1. Waste-Generation Technologies (Metallurgical Process)

The main methods for extracting metallic copper from copper ores are pyrometallurgy and hydrometallurgy, which account for approximately 80–90% and 10–20% of global copper production, respectively [52]. In pyrometallurgical processes, sulphide concentrates are subjected to high-temperature processing, including oxidation, smelting and converting reactions, the products of which are mainly metallurgical slags. Copper slag differs from other metallurgical slags in terms of chemical composition, mineral phases and residual metal content, which gives it significant potential for resource recovery [32]. While ore processing and related hydrometallurgical or gas cleaning processes also generate flotation slurries, sewage sludges, and dusts, this article focuses primarily on pyrometallurgical solid slags, as their massive volume and distinct crystalline matrices represent the primary candidates for anthropogenic deposit classification.

The production of one ton of copper results in the generation of approximately 2.0–3.0 tons of copper slag [52]. Global annual copper production stands at around 17.0 million tons, which translates into 10s of millions of tons of waste each year [53].

Hydrometallurgy involves leaching processes (copper extraction using acid or ammonia), separation (via solvent extraction and back-extraction) and electrodeposition (electrolytic production of cathode copper) [54,55]. Hydrometallurgy is suitable for low-grade ores, but it involves lengthy leaching and separation stages, resulting in low efficiency [39].

In Poland, the largest waste stream consists of flotation tailings, estimated at around 28 million tons per year. The second-largest waste group in terms of volume is metallurgical slag, generated in pyrometallurgical processes, amounting to around 1.1 million tons per year [56]. Tailings from all mines in the Copper Basin are deposited at the “Żelazny Most” landfill, located in Lower Silesia and owned by KGHM Polska Miedź. This landfill is an integral part of the copper production process and constitutes a key link in the production of copper concentrate.

According to Phiri T. Ch. et al. [56], between 1999 and 2019, global copper slag production amounted to 752 million tons across the 13 major copper-producing countries. Furthermore, between 2000 and 2024, global copper slag production in these countries amounted to 966 million tons, with the average annual production estimated at 38.6 million tons [57], with the highest production, exceeding 45 million tons of slag, recorded in 2018 [53]. Chile accounts for the largest share of global copper slag production, having produced 295 million tons over the last 25 years, representing 31% of global production.

#### 2.2.2. Chemical and Phase Composition and Element Occurrence State

The chemical and mineralogical characteristics of copper slag are a direct consequence of the metallurgical process applied, including the type of copper concentrate, the stage of pyrometallurgical processing (smelting, converting or refining), furnace operating conditions, the composition of fluxes, and the cooling regime of the molten material. These technological factors determine not only the bulk chemical composition of the slag but also the occurrence state of valuable and potentially hazardous elements within individual mineral phases. Consequently, the form in which metals occur governs both their technological recoverability and their behavior during subsequent weathering processes. Therefore, the evaluation of copper slag should consider not only its chemical composition but also the mineralogical forms in which individual elements are incorporated [11,44,45,58,59].

Copper slag is highly heterogeneous and chemically diverse. The chemical composition and the proportions of individual mineral phases may vary considerably among different types of slag. The general characteristics of copper slag properties most frequently described in the literature are presented below. The main components of most copper slags include silica (SiO_2_), iron (II) oxide (FeO), iron (III) oxide (Fe_2_O_3_) and small amounts of calcium oxide (CaO) and aluminum oxide (Al_2_O_3_). Depending on their origin, copper slags may also contain metals with recovery potential, such as copper (Cu), cobalt (Co), silver (Ag), nickel (Ni), zinc (Zn) and iron (Fe), as well as potentially toxic elements, including lead (Pb), cadmium (Cd) and arsenic (As), whose presence is of significant importance in terms of environmental impact and slag management [45,53,57,59,60,61,62,63,64]. According to Phiri et al. [44], the concentrations of valuable metals in the slags are high and may include copper (1.19% wt. Cu), cobalt (0.48% wt. Co), silver (1.67% wt. Ag), zinc (1.83% wt. Zn), lead (0.94% wt. Pb), and iron (37.92% wt. Fe), which indicates the significant potential of copper slag as a secondary source of raw materials. Furthermore, Piatak et al. [64] report that the typical composition of copper slag contains 0.42–4.6% Cu, 29–40% Fe, 30–40% SiO_2_, ≤11% CaO, and ≤10% Al_2_O_3_. According to Łabaj et al. [65] the concentration of copper itself is highly dependent on the technological stage of production. In slags from the copper-ore smelting process, the Cu content is 0.5–10% by weight; in slags from the copper-converting process—2–8% by weight; whereas in slags from copper-blistering processes, it may reach as much as 16% by weight [66]. Such compositional variability directly reflects differences in metallurgical technology and determines both the mineralogical composition and the occurrence state of metals within the slag.

The occurrence state of metals represents one of the key characteristics controlling both the technological value and the future behavior of copper slag. Copper in slag produced during smelting occurs primarily in the form of sulphide phases, which account for over 60% of its total content, while smaller amounts are present in the form of oxide phases and metallic copper [67,68]. Copper slags exhibit a diverse range of crystalline phases, amorphous phases, and metallic and sulphide inclusions, which determine their further processing and metal recovery potential. During smelting processes, natural parent minerals undergo complex physicochemical transformations, including volatilization, decomposition, oxidation, reduction and crystallization, resulting in the formation of new phases in the smelting slag [58,69]. The type of mineral phases formed depends on the smelting conditions (temperature and cooling rate) and the chemical composition of the slag. Rapid cooling favors the formation of amorphous phases at the expense of ordered crystalline phases. Slower-cooled slags exhibit a greater variety of phases, which is explained by equilibrium crystallization due to prolonged exposure to smelting conditions [49,59,65]. In turn, the smelting temperature significantly influences the phase composition of the slag, resulting in the formation of various minerals, such as spinels, melilite, olivine, pyroxene and glass, each of which forms at different temperatures and over different time scales [45]. The mineral phases present in slag waste encompass a range of categories, including silicates, oxides, sulphides, pure metals, intermetallic compounds and glass matrices [70]. Their mutual relationships and textural arrangement have been extensively documented using SEM microscopy studies and mineralogical analyses, which enable the identification of individual mineral phases and the occurrence state of valuable metals. The SEM observations reported in [71] demonstrated the co-occurrence of silicate, metallic, and sulphide phases and the presence of copper sulphides trapped in the silicate matrix.

In most fresh copper slags, the dominant crystalline phase is fayalite (Fe_2_SiO_4_), which is most often accompanied by magnetite (Fe_3_O_4_) [45,51,58,64,72,73]. In addition, sulphides are observed, which usually occur as subordinate phases or fine inclusions [71]. The most commonly identified sulphide minerals include chalcocite, chalcopyrite, bornite, galena and sphalerite, while the mineral composition may also include olivine, pyroxene, spinels, hematite, glass, and small amounts of native metals and intermetallic compounds [25,55,58,68].

#### 2.2.3. Environmental Risks

The environmental impact of copper slag is determined not only by its bulk chemical composition but, above all, by the occurrence state of metals within individual mineral phases and their stability under weathering conditions. Although copper slags are generally classified as non-hazardous waste, they contain elevated concentrations of heavy metals and metalloids, including Cu, Zn, Pb, Cd and As, which may become environmentally available depending on the mineralogical form in which they occur and the physicochemical conditions prevailing within the landfill [11,25,45,48,51].

The environmental behavior of copper slag evolves continuously during long-term storage as a result of weathering processes. Primary mineral phases formed during pyrometallurgical processing undergo gradual mineralogical and geochemical transformations, leading to the formation of secondary minerals that may either immobilize or release potentially toxic elements. The extent of these transformations depends on numerous factors, including mineral composition, grain size, oxygen availability, moisture conditions, pH and the composition of infiltrating waters [11,47,58].

Particular attention should be paid to sulphide minerals, which represent the principal occurrence state of copper in fresh slags. Their oxidation may result in the release of metal ions into pore waters, whereas the subsequent precipitation of secondary sulphates, oxides and hydroxides may partially reduce metal mobility by incorporating these elements into newly formed mineral phases [11,25,48]. Consequently, the environmental risk associated with copper slag cannot be evaluated solely on the basis of total metal concentrations but requires detailed mineralogical investigations that identify the occurrence state and stability of individual phases.

A representative example of these long-term transformation processes is provided by the historical copper slag deposited at the Polkowice landfill, described by Zglinicki et al. [47]. In addition to primary pyrometallurgical phases, the slag contains numerous secondary minerals formed during prolonged weathering.

The primary phases include, among others, sulphides: sphalerite [ZnS], wurtzite [(Zn,Fe)S], pyrite [FeS_2_]; sulphates: beaverite-(Zn) [Pb(Fe^3+^Zn)(SO_4_)_2_(OH)_6_] and palmierite [(K,Na)_2_Pb(SO_4_)_2_]; oxides; hydroxides: goethite [Fe^3+^O(OH)], wüstite [FeO], hematite [Fe_2_O_3_], magnetite [Fe^2+^Fe^3+^O_4_]; chromium spinel [Fe^2+^Cr^3+^O_4_]; silicates: petedunnite [Ca(Zn,Mn^2+^,Mg,Fe^2+^)Si_2_O_6_] and quartz [SiO_2_]; and microcline [KAlSi_3_O_8_]. The dominant phase present in the slag, accounting for 5.50–36.30% by weight, is sphalerite. It is accompanied by palmierite (up to 16.5% by weight), beaverite-(Zn) (up to 15.3% by weight) and petedunnite (up to 11.4% by weight). These phases are characterized by a significant degree of oxidation. As a result of long-term transformation processes, secondary sulphate minerals have also formed, namely, basanite Ca(SO_4_)·0.5H_2_O and jarosite KFe^3+^_3_(SO_4_)_2_(OH)_6_, forming surface efflorescences, poorly cemented crusts and pore fillings [47]. These mineralogical transformations clearly demonstrate that the environmental behavior of copper slag changes throughout its storage history and is controlled by progressive alteration of primary phases.

The chemical composition of the Polkowice slag further illustrates the environmental significance of weathered copper slags. The slag consists mainly of SiO_2_ (13.70–20.60% by weight), Fe_2_O_3_ (24.90–39.62% by weight) and, to a lesser extent, CaO (2.71–6.94% by weight), MgO (1.34–4.68% by weight) and Al_2_O_3_ (1.80–3.15% by weight). It is characterized by a high content of Zn (9.42–17.38% by weight), Pb (5.13–13.74% by weight) and Cu (1.29–2.88% by weight). It also contains trace elements, such as Mo (487.4–980.1 ppm), Ni (245.3–530.7 ppm), Sn (2380.0–4441.5 ppm), Sb (2462.8–4446.0 ppm), and Se (168.0–293.0 ppm), and toxic elements, including As (13 100–22 600 ppm) and Cd (190.–893.1 ppm). Although these elements remain largely incorporated within relatively stable mineral phases, changes in environmental conditions may locally enhance their mobility, emphasizing the necessity for long-term monitoring and mineralogical assessment of historical slag deposits.

Overall, the environmental risk associated with copper slag is governed by the interaction between chemical composition, mineralogical occurrence of metals and weathering processes. Therefore, reliable environmental assessment should integrate bulk chemical analyses with detailed mineralogical investigations and leaching studies in order to distinguish metals permanently incorporated into stable silicate or oxide phases from those associated with reactive sulphides or secondary weathering products [11,44,47,48,58].

#### 2.2.4. Resource Utilization

The considerable concentrations of valuable metals retained in copper slags, together with their large volumes generated worldwide, make these materials an important secondary source of mineral resources. Unlike primary ores, copper slags have already undergone high-temperature metallurgical processing, resulting in the enrichment of selected elements within relatively homogeneous waste streams. Consequently, historical slag landfills are increasingly being regarded as anthropogenic deposits that may supplement conventional mineral resources within the framework of a circular economy [44,46,47].

The potential for resource recovery depends not only on the total concentration of valuable metals but also on their occurrence state within the slag. Copper present as metallic inclusions or discrete sulphide phases is generally more accessible for beneficiation and hydrometallurgical processing than copper encapsulated within stable silicate matrices such as fayalite. Therefore, comprehensive mineralogical characterization is an essential prerequisite for selecting appropriate recovery technologies and for evaluating the economic viability of secondary resource exploitation [44,48,58,65].

A representative example of the resource potential of historical copper slags is provided by the Polkowice landfill investigated by Zglinicki et al. [47]. According to these authors, the accumulated waste contains approximately 10,000 tons of Zn, 6700 tons of Pb and 1500 tons of Cu, confirming that long-term deposited metallurgical wastes may constitute economically significant anthropogenic metal deposits. Such deposits represent an important strategic reserve of critical and industrial metals, particularly in regions where the availability of primary mineral resources is gradually declining [47].

In addition to direct metal recovery, copper slags have found numerous applications as secondary mineral raw materials. Driven by the development of sustainable technologies and circular economy strategies, copper slag is increasingly utilized in the cement and construction industries, as well as in various manufacturing applications. In cement production, it serves as a raw material for Portland cement clinker, cement-based composites and as an iron-correcting additive during clinker manufacture [74,75,76,77]. In civil engineering, copper slag is widely used as a substitute for natural aggregates and sand in concrete production, as well as in the manufacture of bricks and precast building elements [77,78].

The diversification of possible utilization pathways significantly reduces the amount of material requiring long-term disposal while simultaneously limiting the consumption of primary raw materials. However, the selection of an appropriate management strategy should always be preceded by detailed chemical and mineralogical characterization, since the technological performance and environmental safety of copper slag are controlled by its phase composition and the occurrence state of individual elements [11,44,58].

Overall, the contemporary approach to copper slag management extends beyond its classification as industrial waste. Owing to its substantial content of valuable metals, favorable mineralogical characteristics and wide range of potential industrial applications, copper slag should increasingly be regarded as an anthropogenic deposit and an integral component of sustainable resource management. The combination of selective metal recovery with material reuse offers an effective route towards implementing circular economy principles while simultaneously reducing the environmental footprint of historical and newly generated metallurgical waste landfills.

### 2.3. Iron and Steel Metallurgy Waste

The processing of iron ores and the production of steel and cast steel constitute a fundamental pillar of the global metallurgical industry, while simultaneously generating the largest stream of solid industrial waste by mass [79,80]. The main waste product of this sector is metallurgical slag, which, based on the stage of the technological process and the type of production unit, is classified into two basic groups [81,82,83], as follows:

Blast furnace slags (BFSs), generated during the reduction of iron ores and the production of blast furnace pig iron [65,84].

Steelmaking slags (converter, electric, open-hearth), generated during the direct smelting and refining of steel and cast steel in electric arc furnaces (EAFs) or basic oxygen furnaces (BOFs) [85,86].

#### 2.3.1. Waste-Generation Technologies (Metallurgical Process)

The technological methods and processes used in iron and steel production directly determine the type, volume, and physicochemical properties of the resulting slag [2,87].

Blast furnace slag (BFS) is formed in a blast furnace during the reduction of iron ores with coke in the presence of fluxes (primarily limestone or dolomite) at temperatures in the range of 1400–1500 °C [88]. On a global scale, blast furnace slag production is estimated at 220 to 370 kg per each ton of produced pig iron [4,80,84].

On the other hand, steelmaking slags—including Linz–Donawitz converter slag (LD converter slag) and electric arc furnace slag (EAF slag), which are generated predominantly during the processing of steel scrap—are characterized by a unit production level ranging from 100 to 150 kg per ton of crude steel [89,90].

On a global scale, the annual generation of slag from iron and steel metallurgy is estimated to be between 560 and 720 million tons, which many times exceeds the volume of waste from non-ferrous metallurgy [4,35]. Based on technological waste-generation proportions recognized in the literature [50,80], it is estimated that with current global pig iron production exceeding 1.4 billion tons per year, blast furnace waste accounts for approximately 250–320 million tons, while the annual mass of steelmaking slags reaches the range of 200–290 million tons.

Under domestic conditions (Poland), the annual production of slag from pyrometallurgical iron and steel processes amounts to approximately 1.1–1.3 million tons [91]. Waste deposited in landfills, especially of older generations, undergoes continuous natural weathering, which leads to significant transformations of its phase and chemical composition and also poses specific environmental risks [18,65,85]. Under regional conditions (Upper Silesia), these processes are directly reflected in local geochemical anomalies of soils and surface sediments (e.g., elevated concentrations of heavy metals and metalloids, such as Pb, Zn, Cd, and As) around historical storage sites [92].

In both European and national legislation, these wastes—in accordance with the Waste Catalogue—are classified under group 10 (wastes from thermal processes). In most cases, iron and steelmaking slags are classified as non-hazardous waste. Due to the enormous quantities of their historical deposition, combined with a high content of useful mineral and metallic phases, these landfills are currently being intensively investigated and redefined as valuable anthropogenic deposits with significant resource potential for road construction, the cement industry, and civil engineering [82,93,94,95,96].

#### 2.3.2. Chemical and Phase Composition and Element Occurrence State

The chemical and structural properties of iron and steelmaking slags exhibit high variability, determined by the type of feed (grade of ore, inclusions, and quality of steel scrap and alloying additives), the type of fluxes used, and the specific dynamics of cooling the liquid melt [84,85,96]. In terms of the chemical composition, the following four main oxide components dominate in blast furnace and steelmaking slags: calcium oxide (CaO), silicon dioxide (SiO_2_), aluminum oxide (Al_2_O_3_), and iron oxides (iron (II) oxide—FeO and iron (III) oxide—Fe_2_O_3_). A crucial technological and environmental parameter of iron slags is their basicity index, defined as the ratio of calcium oxide content to silicon dioxide (Z = CaO/SiO_2_).

Blast furnace slags are characterized by high contents of silicon dioxide (SiO_2_) (30–40%) and calcium oxide (CaO) (35–45%), with a relatively low total iron content (<1–2%) [91,94]. This system is classified as calcium–silicate with significant contents of aluminum oxide (Al_2_O_3_) (5–15%) and magnesium oxide (MgO) (1–12%) [88].

Steelmaking slags are distinguished from blast furnace slags by a significantly higher basicity (Z > 2–3), a calcium oxide (CaO) content at the level of 40–55%, and a substantial share of iron oxides (FeO and Fe_2_O_3_, combined within 10–25%, reaching up to 30% in specific phases of basic oxygen converters), as well as manganese oxide (MnO, within 1–8%) and phosphorus (V) oxide (P_2_O_5_) [79,86]. This high concentration of free CaO and Fe is a critical issue, as it limits direct applications and requires specific processing. It is important to emphasize that the steel slag composition strongly depends on the elaboration process type. Slags generated from alkaline (basic) processes (e.g., BOF or EAF with basic refractory linings) are characterized predominantly by highly basic minerals such as dicalcium and tricalcium silicates, alongside free lime (CaO), periclase (MgO), wüstite, and complex calcium ferrites. In contrast, slags from acid processes contain mostly iron-rich silicates, such as fayalite and rhodonite, due to the different fluxes and siliceous refractory materials used.

The phase and mineralogical composition of these wastes determines their susceptibility to weathering and environmental stability [96]. The structure of iron and steelmaking slags distinguishes between a glassy matrix (amorphous substance) and crystalline phases [95].

The main silicate and aluminosilicate phases: Silicates of calcium and magnesium dominate in slags. Under conditions of the slow air-cooling of blast furnace slag, melilites crystallize predominantly—including gehlenite (Ca_2_Al[AlSiO_7_]) and akermanite (Ca_2_Mg[Si_2_O_7_])—as well as merwinite (Ca_3_Mg(SiO_4_)_2_), pyroxenes (diopside and augite), anorthite (CaAl_2_Si_2_O_8_), and olivines (fayalite (Fe_2_SiO_4_) and forsterite (Mg_2_SiO_4_)). An important component of highly basic steelmaking slags are dicalcium silicates (larnite, including β-Ca_2_SiO_4_ and γ-Ca_2_SiO_4_ modifications) and tricalcium silicates (alite (Ca_3_SiO_5_)). Calcium orthosilicate, due to polymorphic transformations occurring at temperatures of 440–510 °C, exhibits a tendency to change in volume, which can cause unfavorable disintegration and spontaneous dusting of the aggregate [97,98].

Oxide phases and spinels: These are represented mainly by magnetite (Fe_3_O_4_), hematite (Fe_2_O_3_), and wüstite (FeO), as well as complex calcium ferrites (e.g., Ca_2_Fe_2_O_5_) and solid solutions of divalent metal oxides, e.g., RO (where R = Mg, Fe, and Mn). Of particular importance is the presence of free lime (CaO) and free magnesia (periclase (MgO)), the hydration of which leads to the formation of hydroxides. This process induces swelling of the material (even by up to a dozen percent by volume), which limits direct construction applications and necessitates natural weathering, seasoning, or accelerated carbonation of the heaps [86]. Furthermore, the hydration of free lime significantly raises the pH of leachates (often exceeding pH 11–12), which poses a risk of contaminating groundwater by mobilizing amphoteric metals and metalloids. Mitigating this negative effect is key when designing volume-stable blended cements with the addition of steelmaking slags [99,100].

Metallic inclusions: a characteristic feature of older slags deposited on heaps is the presence of spherical extractions of pure metallic iron (Fe) and alloy phases (cast iron and steel), which were not precisely separated during historical furnace tapping and currently serve as a target for secondary magnetic recovery [91].

In the case of rapid cooling (e.g., granulation of liquid blast furnace slag with water), the structure of the material is vitrified into a high content of silicate glass—resulting in granulated blast furnace slag (GBFS). This imparts excellent latent-hydraulic properties to these wastes, making them widely utilized in the cement industry as a clinker substitute and a valuable component of composite mineral binders [81,93].

#### 2.3.3. Environmental Risks

The environmental safety and stability of basic steelmaking slags are strongly dependent on the occurrence of highly reactive phases. Of particular importance is the presence of free lime (CaO) and free magnesia (periclase (MgO)), the hydration of which leads to the formation of hydroxides. This process induces swelling of the material (even by up to a dozen percent by volume), which poses physical instability risks and necessitates natural weathering, seasoning, or accelerated carbonation of the heaps. Furthermore, the hydration of free lime significantly raises the pH of leachates (often exceeding pH 11–12), which poses a risk of contaminating groundwater by mobilizing amphoteric metals and metalloids. Mitigating this negative effect is key when designing volume-stable blended cements with the addition of steelmaking slags [100].

#### 2.3.4. Resource Utilization

Recognizing waste storage sites from the thermal processing of iron and steel (blast furnace and steelmaking slags) as anthropogenic deposits requires a precise estimation of their geological–economic balance resources. The primary barrier to classical resource balancing of iron metallurgy heaps is their extreme spatial heterogeneity and the chaotic manner of material deposition within the dump structure. This directly stems from historical technological changes in operating plants, irregular emptying of loads, and the mixing of waste streams from different production units [85,95].

Balance resources of iron metallurgy heaps are estimated using geometric–statistical methods (mainly the block method and the arithmetic mean method), dividing the landfill body into geometric calculation domains defined by the dump morphology or the distribution range of specific slag fractions [101]. In the case of anthropogenic iron and steel deposits, procedures for balancing qualitative and quantitative parameters focus on two fundamental aspects.

Metallic fraction resources (useful fraction): Old metallurgical dumps contain significant amounts of so-called tappings and inclusions of pure iron or high-percentage steel scrap that became trapped in the slag structure during the tapping of liquid material from the furnace. The metallic iron (Fe_met) content in certain zones of the dumps can fluctuate from a few to even a dozen mass percent. This provides a direct basis for isolating independent calculation blocks characterized by high economic viability for secondary mining extraction using industrial magnetic separation [51,102].

Aggregate and binder resources (accompanying raw material): The remaining slag mass (silicate–aluminosilicate matrix), which constitutes the overburden or geological background for metallic inclusions, is balanced as an anthropogenic deposit of artificial aggregates or raw materials for the cement industry. Its balance suitability and resource qualification are determined primarily by mineral stability—the presence of free lime (CaO) and the inherent tendency toward silicate disintegration of steelmaking slags dictate and often limit the possibility of classifying certain parts of the dump as industrial resources [95,98]. Proper selection and stability of this material are crucial for the subsequent durability of concrete composites manufactured using such aggregates [102]. It is also noteworthy that blast furnace slag is a highly valuable raw material for geopolymer production; utilizing these slags in alkali-activated binders constitutes an important sustainable approach that significantly reduces the carbon footprint.

The inclusion of anthropogenic blast furnace and steelmaking slag resources into the national economic balance partially compensates for the complete lack of natural iron ore deposits with industrial parameters in Poland (which were definitively removed from the national resource balance in 1994).

## 3. Discussion

### 3.1. Chemical and Phase Heterogeneity as a Driver of Resource Potential

A detailed characterization of the chemical and phase compositions of various slags (as presented in Section 2) is a fundamental prerequisite for evaluating their potential as anthropogenic deposits. A comparison of the main oxides and mineral phases reveals whether valuable metals (Cu, Pb, Zn, and Fe) are trapped within stable silicate and aluminosilicate matrices (e.g., fayalite or melilites) or present as separate, highly reactive sulphide, oxide, or metallic inclusions. This speciation directly governs the technological feasibility and economic viability of their extraction. For instance, high concentrations of Zn and Pb in easily weatherable sulphate or sulphide phases in Zn-Pb slags present both an acute environmental risk and an immediate target for selective metallurgical recovery. In contrast, the recovery of iron from steelmaking slags relies on isolating distinct metallic inclusions from the calcium–silicate matrix. Thus, understanding the mineralogy is the first step in geometric–statistical resource balancing.

### 3.2. Comparative Analysis of Metallurgical Waste Deposits

A comparison and evaluation of the chemical composition of metallurgical slags shows deep variation depending on the type of processed ore and the specifics of the pyrometallurgical processes [15,18,35,48,91]. A detailed, comparative compilation of the main oxide components and key accompanying metals for zinc–lead, copper, blast furnace, and steelmaking slags is presented in Table 1. It allows for the direct identification of the differences in material basicity and the concentrations of useful elements [44,47,65,79,86,95,98].

The consequence of this chemical diversification is a highly distinct mineralogical profile of the studied wastes, determining their subsequent environmental stability and economic utility [15,18,35,45,48,95]. In Table 2, a synthetic division and classification of the dominant mineral and crystalline phases into the following three main groups are presented: silicates/aluminosilicates, oxides/spinels, and sulphides/sulphates. Such a framework allows for the accurate prediction of the behavior of slag materials under long-term landfill deposition conditions [2,62,86,96].

Reliable forecasting of the long-term environmental stability of slags is based on integrating micromineralogical studies with thermodynamic modeling and standardized leaching tests. It is precisely the identification of the occurrence form of elements—e.g., their permanent entrapment within weathering-resistant oxides and glassy matrix, as opposed to highly reactive sulphide phases or hydrating free lime—that allows for a reliable assessment of the risk of contaminant release into the groundwater environment.

### 3.3. Resource Potential in the Context of Anthropogenic Deposits

To provide a cohesive evaluation of the resource potential discussed in the preceding sections, the estimated reserves of selected strategic metals (Zn, Pb, Cu, Fe) from documented Polish metallurgical landfills have been synthesized. Table 3 presents a comparative overview of the specific resource assumptions for domestic Zn-Pb, Cu, and Fe/steel slag deposits, demonstrating their significant role as secondary raw material bases.

Despite the vast resource potential of the metallurgical waste residues discussed above, actual development and recovery face significant economic and technical limitations that depend heavily on the occurrence state of the elements. In iron and steelmaking dumps, macroscopic metallic iron inclusions can be effectively and profitably recovered using magnetic separation. However, the recovery of metals chemically bound within a silicate–calcium matrix (e.g., iron in wüstite or calcium ferrites) is currently limited by high energy requirements and prohibitive recovery costs. The economic viability of utilizing the remaining mineral matrix is further constrained by environmental requirements, particularly the need for costly pretreatment (e.g., accelerated carbonation) to neutralize the hydration risks of free lime in steel slags and ensure their volume stability.

One factor in favor of processing zinc–lead smelting slag is its potential negative impact on the environment, due to its significant metal content, including toxic metals.

The mobility of metals and metalloids in the soil–water environment is determined primarily by the form (chemical and mineral composition) in which they are introduced into this environment along with waste, as well as by environmental conditions, i.e., precipitation, temperature, Eh potential, and pH.

As mentioned earlier, one of the factors determining the solubility of components present in slag and their mobility in the environment is their mineralogical and chemical composition. It has been found that silicate and spinel phases are the most resistant to weathering processes, whereas sulphide, glassy, and intermetallic phases [2,14] exhibit significantly higher solubility in the soil–water environment.

Given the phase composition of Zn-Pb smelting slags, which are dominated by polyphase silicate and oxide conglomerates, pyrometallurgical processing is the most effective method for treating them [8].

Due to the complexity of the mineral composition of the studied metallurgical wastes, which are predominantly multiphase conglomerates, it is difficult to unambiguously determine the mobility of the elements they contain in the environment [2,8].

Copper slags provide a representative example of the complex relationship between element occurrence state, environmental behavior and resource recovery. Although their total metal content is often sufficiently high to justify their classification as anthropogenic deposits, economic recovery cannot be assessed solely on the basis of bulk chemical composition. The mineralogical occurrence of metals largely determines both their environmental stability and the feasibility of extraction. Copper enclosed within fayalite or glassy matrices exhibits relatively low mobility under natural weathering conditions, which generally reduces the environmental risk but simultaneously makes metallurgical recovery more difficult and energy intensive. In contrast, copper present as sulphide or metallic inclusions is considerably more accessible for flotation or hydrometallurgical processing but may also represent the fraction most susceptible to weathering and metal release. This illustrates the fundamental trade-off between environmental stability and resource recoverability, which should be considered during the evaluation of anthropogenic deposits.

Despite the considerable resource potential of historical copper slag landfills, several technical bottlenecks continue to limit their large-scale utilization. One of the major challenges is the strong mineralogical heterogeneity of deposited materials resulting from changes in metallurgical technologies, feed composition and cooling conditions over decades of operation. As a consequence, individual landfill zones may differ significantly in both metal content and mineralogical composition, making resource estimation and process optimization difficult. Furthermore, valuable metals are frequently finely disseminated within silicate matrices, reducing the efficiency of conventional physical beneficiation methods. Future development of anthropogenic mining, therefore, requires integrated geological, mineralogical and metallurgical characterization supported by modern geometallurgical approaches capable of linking resource quality with appropriate recovery technologies.

The transition from waste landfill to anthropogenic deposit requires not only sufficient metal concentrations but also detailed knowledge of element occurrence state, mineralogical heterogeneity and long-term environmental stability.

Although this relationship is particularly evident in copper slags, the same principle applies to other metallurgical wastes discussed in this review. Regardless of the type of slag, the occurrence state of valuable and potentially hazardous elements represents the key factor linking environmental performance with the feasibility of secondary resource recovery.

**Figure 1:**
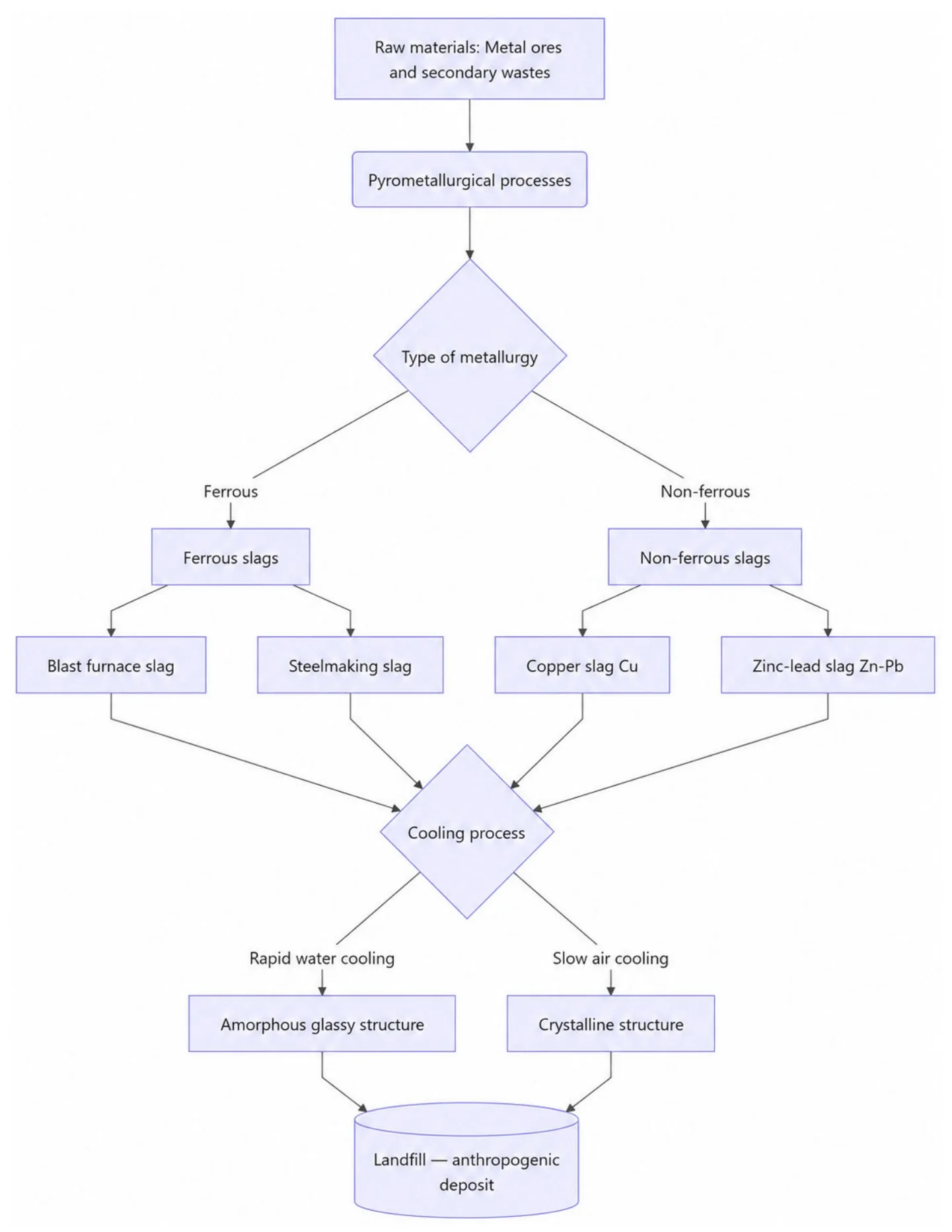
Schematic diagram illustrating the formation process of metallurgical slags.

To synthesize the interrelations explored in this review, comprehensive schematic diagrams have been developed to reflect the domain’s patterns systematically. The formation processes of metallurgical slags are visualized in Figure 1, mapping the pathways from raw materials to final deposit structures based on technological choices and cooling methods.

**Figure 2:**
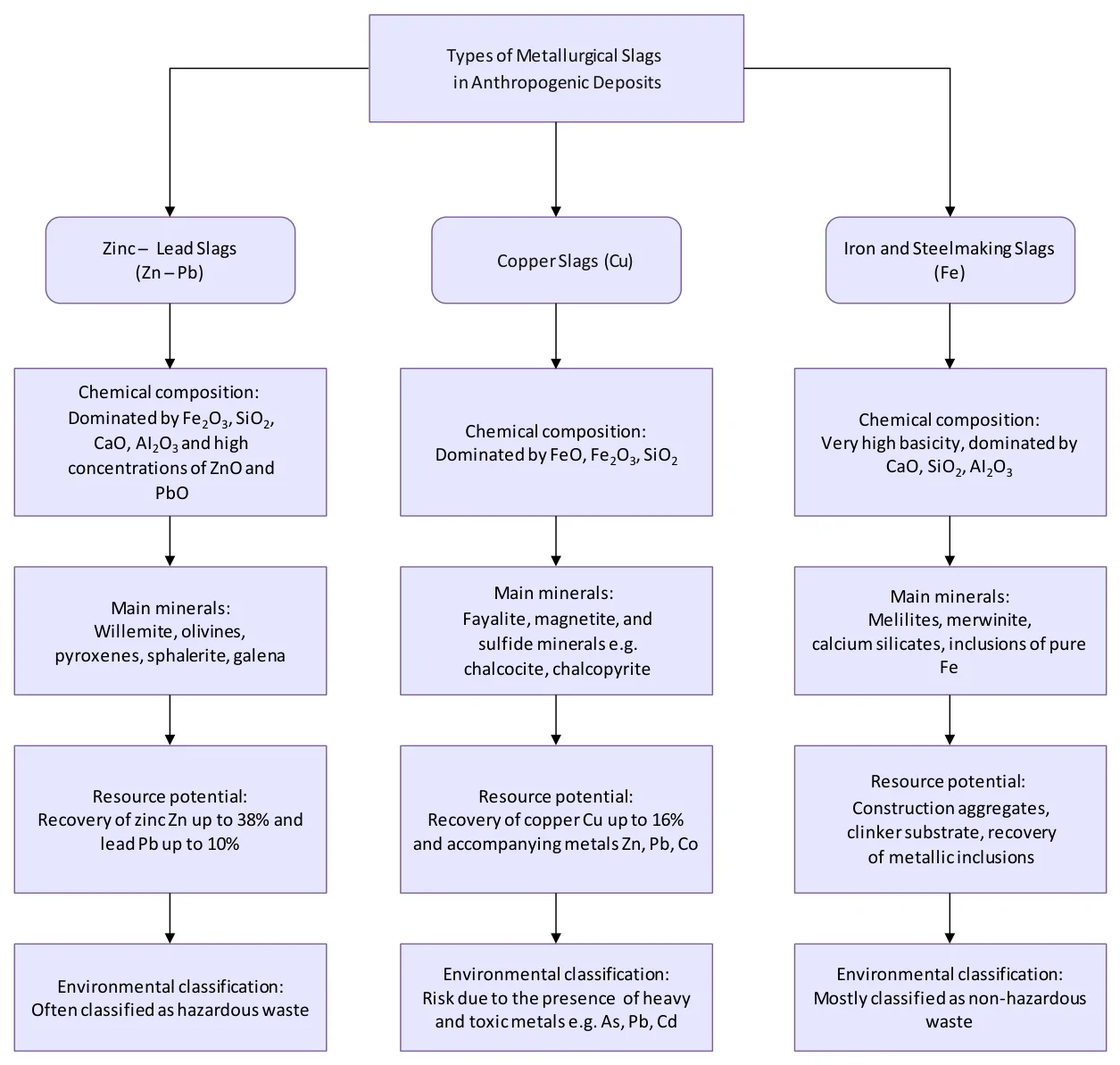
Comparison of the characteristics of different types of slags (Zn-Pb, Cu, Fe).

A detailed comparison of the characteristics of different types of slag, highlighting the chemical compositions, main minerals, and resource potential, is presented in Figure 2.

**Figure 3:**
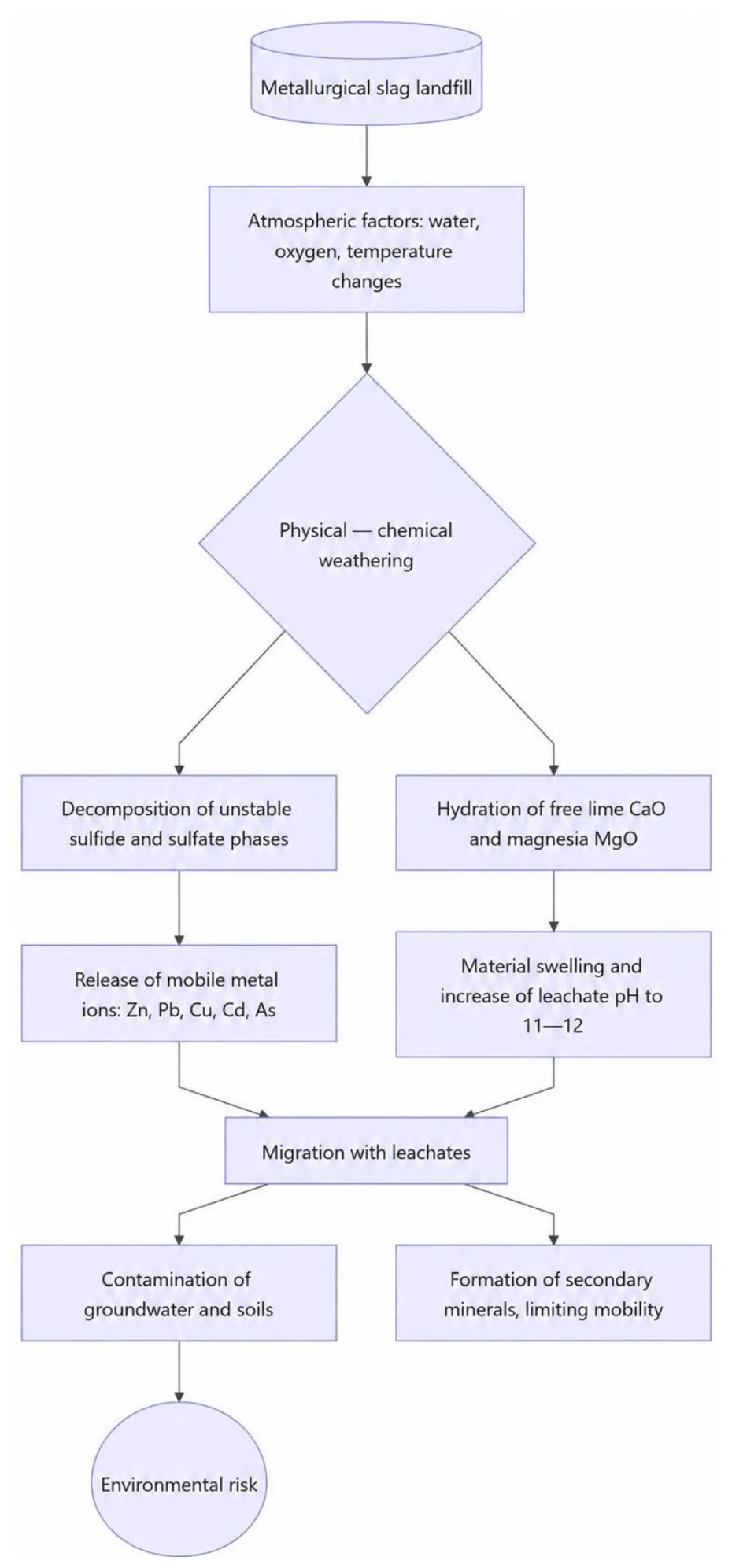
Mechanism of the environmental risks associated with metallurgical slag landfills.

The mechanism of the environmental risks, specifically addressing weathering, phase decomposition, and contaminant migration, is illustrated in Figure 3.

**Figure 4:**
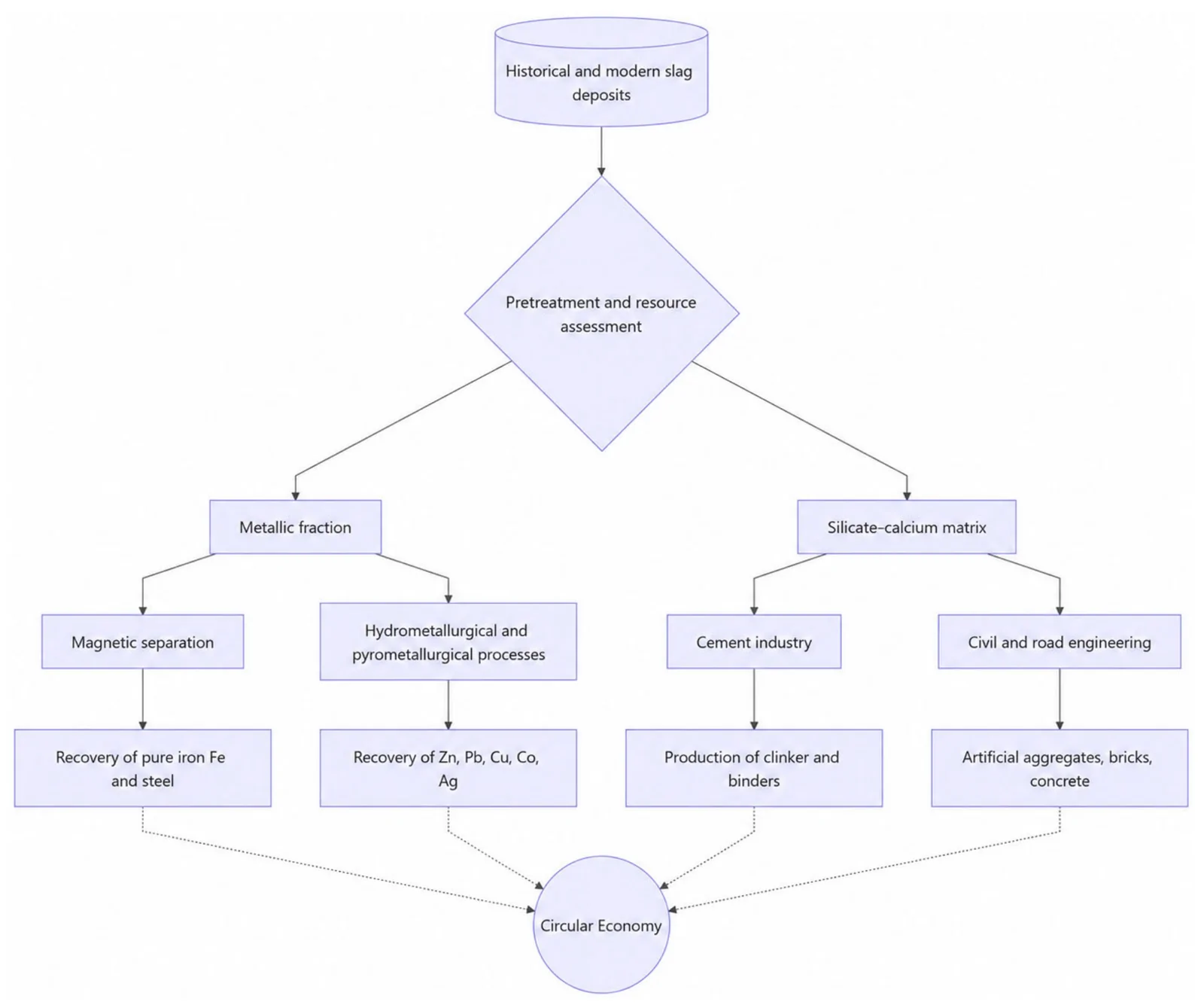
Utilization routes for resources from metallurgical slag deposits within the circular economy.

Furthermore, Figure 4 maps the utilization routes for resources from metallurgical slag deposits within the circular economy framework, integrating pretreatment, metallic fraction recovery, and mineral matrix utilization.

The comparison presented in Table 4 demonstrates that the economic value of metallurgical slags cannot be assessed solely based on total metal concentrations. Instead, their resource potential is controlled by the occurrence state of valuable elements, mineralogical composition, environmental stability and the availability of appropriate recovery technologies.

The economic feasibility of recovering metals from anthropogenic deposits cannot be evaluated solely on the basis of their total concentrations. Although historical metallurgical slags often contain economically significant quantities of valuable elements, the profitability of recovery is strongly influenced by their occurrence state, mineralogical associations and spatial distribution within the landfill. Metals finely disseminated within stable silicate matrices generally require more complex and energy-intensive processing than those occurring as discrete metallic or sulphide phases. Consequently, recovery costs are closely related to mineralogical characteristics and the degree of liberation of valuable phases. In addition, environmental constraints associated with waste excavation, processing and long-term landfill management must be considered when selecting appropriate recovery technologies. Therefore, the sustainable development of anthropogenic deposits requires balancing economic viability with environmental protection, emphasizing the importance of integrated geological, mineralogical and technological assessment.

Future research should, therefore, focus not only on improving metal recovery technologies but also on developing integrated assessment methodologies that simultaneously consider resource quality, recovery efficiency, environmental performance and economic feasibility.

## 4. Conclusions

Due to their high metal content, metallurgical slags can be a valuable secondary raw material, and the landfills in which they are deposited can constitute anthropogenic deposits.

The chemical composition of slag from Zn-Pb smelting is dominated by Fe_2_O_3_, SiO_2_, CaO, Al_2_O_3_, ZnO and PbO, with their concentrations varying widely from a few to as many as several dozen %.

The phase composition of these slags is highly varied; alongside monophasic components, there are a number of polyphasic grains, consisting of intergrown clusters of individual phases, one of which is always dominant.

The slags produced during lead refining are characterized by high concentrations of metals and metalloids, which are not found in slags from the Zn-Pb shaft process; therefore, the landfill site in which they are selectively deposited can be regarded as an anthropogenic deposit.

Waste from copper smelting, particularly the slag produced during pyrometallurgical processes, accounts for a high proportion of solid waste in the non-ferrous metals industry. Smelting technologies generate approximately 2–3 tons of slag waste per ton of copper produced, which on a global scale amounts to tens of millions of tons per year.

The chemical composition of slag is complex and depends on the raw material and the technology used; the predominant components are iron and silicon oxides, as well as metals such as Zn, Pb, Cu, and Cr. The presence of heavy metals and toxic elements poses challenges for the industry in terms of the safe storage and management of this waste.

Given their content of valuable metals and the large volume of waste involved, copper smelting slags can be regarded as anthropogenic raw material deposits, offering opportunities for their secondary recovery and reuse. Incorporating such waste into circular economy strategies can not only reduce pressure on primary resources but also limit environmental impact.

In contrast to non-ferrous metallurgy, the processing of iron ores and the production of steel and cast steel generate the largest stream of solid thermal waste by mass on a global scale, reaching an annual volume of 560–720 million tons. The comparative analysis performed indicates that although unit waste indicators for copper metallurgy are significantly higher (2.0–3.0 t of slag/t of metal) than for iron and steel metallurgy (where the indicator for blast furnace slags is a maximum of 0.37 t/t of pig iron), the mass dominance of the latter sector uniquely determines the structure of industrial landfills worldwide and in Poland.

The chemical and mineralogical profile of iron and steel slags shows fundamental discrepancies compared with heavy metal slags. While in Zn-Pb and Cu slags the key element being balanced is the concentration of sulphide and oxide phases of valuable accompanying metals (such as copper, zinc, or lead) representing potential for secondary recovery, in the case of iron metallurgy, the basis for delineating calculation blocks is the resource of the metallic fraction (inclusions of pure iron and steel scrap) subject to magnetic separation.

In turn, the remaining silicate–calcium matrix of these slags, characterized by high basicity and the presence of phases such as melilites or di- and tricalcium silicates, redefines these landfills as valuable deposits of accompanying raw materials. Due to their hydraulic properties (in the case of rapid cooling) or suitability as artificial aggregates, they represent a strategic alternative to natural raw materials in construction, road engineering, and the cement industry.

The integration of anthropogenic slag resources from iron and steel metallurgy into the national economic balance also carries a historical–strategic dimension. Under Polish conditions, it allows for partial compensation for the total lack of natural, industrial iron ore deposits, which were permanently removed from the national resource balance at the end of the 20th century.

Due to the current circular economy policy, the future of the raw materials industry and environmental engineering is moving toward the extraction of elements from artificial deposits (industrial waste). To this end, extensive research is being conducted on modern methods for processing metallurgical waste, primarily slag, such as biohydrometallurgical methods using bacteria.

Despite its high efficiency, bioleaching is currently being conducted only on a laboratory scale; due to the long duration of the process, low yield, and difficulties in separating metals from the solution, it has not yet found application on an industrial scale.

Three-dimensional modeling is increasingly being used (currently in pilot studies) to determine resource abundance.

In conclusion, regardless of the metallurgical sector, modern management of post-metallurgical heaps requires treating them as evolving anthropogenic deposits. However, the successful implementation of circular economy principles and the determination of the full economic utility of the analyzed facilities necessitate the continuous development of optimal and environmentally sustainable methods for extracting both useful metallic phases and the accompanying mineral matrices.

## Figures and Tables

**Table 1:** Comparative summary of the main chemical compositions of the slags [wt. %].

Component	Zinc–Lead Slags (Zn-Pb) (General/Domestic)	Copper Slags (Cu) (General/Domestic)	Blast Furnace Slags (Fe)	Steelmaking Slags (Fe)	References
CaO	0.18–32.23%/2.0–25.0%	≤11.0%/2.71–6.94%	35.0–45.0%	40.0–55.0%	[15,16,17,18,47,65,86,95]
SiO_2_	2.04–57.10%/10.0–40.0%	30.0–40.0%/13.70–20.60%	30.0–40.0%	10.0–20.0%	[2,15,47,65,98]
Al_2_O_3_	0.90–21.90%/1.5–15.0%	≤10.0%/1.80–3.15%	5.0–15.0%	Minor share	[16,48,88,95]
MgO	0.61–15.90%/1.0–10.0%	1.0–5.0%/1.34–4.68%	1.0–12.0%	3.0–10.0%	[48,79,86,98]
Fe_total (incl. FeO/Fe_2_O_3_)	0.88–59.60%/15.0–45.0%	29.0–40.0%/24.90–39.62%	<1.0–2.0%	10.0–25.0%	[47,64,79,95]
Sulphur (S)	0.5–6.0%	0.2–3.5%	<2.0%	<0.5%	[15,44,86,95]
Main accompanying metals	Zn: 0.02–38.0% (gen.)/5.0–15.0% (dom.) Pb: 0.0002–5.8% (gen.)/1.0–10.0% (dom.)	Cu: 0.42–16.0% (gen.)/1.29–2.88% (dom.) Zn: ~1.83% (gen.)/9.42–17.38% (dom.) Pb: ~0.94% (gen.)/5.13–13.74% (dom.)	Trace amounts	MnO: 1.0–8.0% P_2_O_5_: trace	[2,17,44,47,65,66,79,103]

**Table 2:** Synthesis of the dominant mineral and crystalline phases.

Mineral Group	Zinc and Lead Metallurgy (Zn-Pb)	Copper Metallurgy (Cu)	Iron and Steel Metallurgy (Fe)
Silicates and aluminosilicates	Willemite (Zn_2_SiO_4_); olivines (fayalite (Fe_2_SiO_4_), kirschsteinite (CaFe^2+^SiO_4_), forsterite (Mg_2_SiO_4_)); pyroxenes (XY(Si,Al)_2_O_6_); melilites ((Ca,Na)_2_(Al,Mg)[(Si,Al)_2_O_7_])	Fayalite (Fe_2_SiO_4_), pyroxenes, petedunnite (Ca(Zn,Mn,Mg,Fe)Si_2_O_6_), microcline (KAlSi_3_O_8_), quartz (SiO_2_)	Melilites (gehlenite (Ca_2_Al[AlSiO_7_]), akermanite (Ca_2_Mg[Si_2_O_7_])); merwinite (Ca_3_Mg(SiO_4_)_2_); anorthite (CaAl_2_Si_2_O_8_); dicalcium and tricalcium silicates (larnite (Ca_2_SiO_4_), alite (Ca_3_SiO_5_)); pyroxenes (diopside, augite); olivines (fayalite (Fe_2_SiO_4_), forsterite (Mg_2_SiO_4_))
Oxides and spinels	Zincite (ZnO); wüstite (FeO); hematite (Fe_2_O_3_); goethite (FeO(OH)); spinels (magnetite (Fe_3_O_4_), hercynite (FeAl_2_O_4_), franklinite (ZnFe_2_O_4_), gahnite (ZnAl_2_O_4_), ulvöspinel (Fe_2_TiO_4_))	Magnetite (Fe_3_O_4_), hematite (Fe_2_O_3_), goethite (FeO(OH)), wüstite (FeO), chrome spinel (FeCr_2_O_4_)	Magnetite (Fe_3_O_4_), hematite (Fe_2_O_3_), wüstite (FeO), calcium ferrites (Ca_2_Fe_2_O_5_), periclase (MgO), free lime (CaO)
Sulphides and sulphates	Sphalerite (ZnS); galena (PbS); pyrite (FeS_2_); pyrrhotite (FeS); digenite (Cu_9_S_5_); cubanite (CuFe_2_S_3_); covellite (CuS); chalcocite (Cu_2_S); sulphates (anglesite (PbSO_4_), goslarite (ZnSO_4_·7H_2_O), gypsum (CaSO_4_·2H_2_O), ktenasite (ZnCu_4_(SO_4_)_2_(OH)_6_·6H_2_O), posnjakite (Cu_4_[(OH)_6_SO_4_]·H_2_O))	Sphalerite (ZnS); wurtzite ((Zn,Fe)S); pyrite (FeS_2_); chalcocite (Cu_2_S); chalcopyrite (CuFeS_2_); bornite (Cu_5_FeS_4_); galena (PbS); arsenopyrite (FeAsS); beaverite-(Zn) (Pb(Fe,Zn)_3_(SO_4_)_2_(OH)_6_); palmierite ((K,Na)_2_Pb(SO_4_)_2_); secondary sulphates (basanite (CaSO_4_·0.5H_2_O), jarosite (KFe_3_(SO_4_)_2_(OH)_6_)	Do not occur as dominant phases

**Table 3:** Estimated resource potential of selected metals in major Polish metallurgical slag landfills (based on domestic primary research and documentation).

Deposit Type (Location in Poland)	Estimated Total Slag Mass/Volume	Zn Resources/Content	Pb Resources/Content	Cu Resources/Content	Fe (Metallic) Resources/Content	Reference(s)
Zn-Pb Refining Slag Landfill (Case Study—Section 3.3)	~0.6 ha (Specific refining slag zone)	2856 Mg	5206 Mg	-	-	[8]
Historical Zn-Pb Smelting Dumps (Upper Silesia & Olkusz Region, e.g., Bytom, Piekary, Sławków, Świętochłowice)	Historically accumulated heaps (Millions of tons)	High variability: up to 13.9 wt.%	High variability: up to 3.9 wt.%	Minor (<1.0 wt.%)	Bound in spinels and silicates	[12,22,27,29,33,34]
Copper Slag Tailings Dump (Polkowice Site)	Total historically accumulated mass at the specific dump	~10,000 Mg	~6700 Mg	~1500 Mg	Variable (bound in fayalite/magnetite)	[47]
Historical & Modern Cu Slags (Old Copper Basin, SW Poland & KGHM Operations)	Historical local heaps + current national generation (~1.1 mln t/year)	0.1–0.5 wt.%	0.1–0.3 wt.%	up to 2.5 wt.% (historical) to 10.0 wt.% (smelting)	10.0–45.0 wt.% (total Fe)	[25,49,50,56,103]
Iron & Steelmaking Dumps (Upper Silesia Region)	~1.1–1.3 million tons/year (national generation) + massive historical heaps	Traces	Traces	Traces	Significant high-percentage metallic tappings (Fe_met up to 10–15 wt.%)	[7,50,85,91,95]

This table synthesizes data from multiple domestic studies to present the resource potential of Polish metallurgical slag deposits. For fully documented specific zones (e.g., the refining landfill [8] and the Polkowice dump [47]), absolute resource estimates are provided in Megagrams [Mg]. For historical regional heaps across Upper Silesia, the Olkusz region, and the Old Copper Basin, the potential is expressed as the maximal weight percentage concentration [wt.%] identified in the respective literature. Estimates for recoverable metallic iron inclusions in domestic steelmaking dumps are based on [47,102], with regional characterization supported by [85,87,95].

**Table 4:** Integrated comparison of metallurgical slags in terms of resource potential and environmental performance.

Feature	Zn–Pb Slags	Copper Slags	Iron & Steel Slags
Main resource elements	Zn, Pb, Cu	Cu, Fe, Co, Ag, Ni	Fe
Dominant occurrence state	Silicates, oxides, sulphides, metallic phases	Sulphides, fayalite, magnetite	Metallic Fe, oxides, calcium silicates
Main environmental concern	Zn, Pb leaching	Weathering of sulphides, As, Cd	Free CaO, high alkalinity
Major technical bottleneck	Complex multiphase intergrowths	Copper enclosed in silicates	Separation of metallic Fe, free lime
Most suitable recovery route	Pyrometallurgy, biohydometallurgy	Flotation + hydrometallurgy + pyrometallurgy	Magnetic separation + aggregate recovery
Main material utilization	Metal recovery	Cement, aggregates, abrasives	Cement, road construction, aggregates
Anthropogenic deposit potential	High	High	Moderate–High

## Data Availability

No new data were created or analyzed in this study. Data sharing is not applicable to this article.
